# Plant leaves for wrapping *zongzi* in China: an ethnobotanical study

**DOI:** 10.1186/s13002-019-0339-7

**Published:** 2019-12-11

**Authors:** Fengke Lin, Binsheng Luo, Bo Long, Chunlin Long

**Affiliations:** 1 Key Laboratory of Ethnomedicine (Minzu University of China), Ministry of Education, Beijing, 100081 China; 2 College of Life and Environmental Sciences, Minzu University of China, Beijing, 100081 China; 3 School of Life Science, Yunnan University, Kunming, 650091 China; 4 Kunming Institute of Botany, Chinese Academy of Sciences, Kunming, 650201 China

**Keywords:** Plant leaves, *zongzi*, Dragon Boat Festival, Traditional botanical knowledge, Chinese symbolic food

## Abstract

**Background:**

*Zongzi*, a common Chinese rice-pudding and one of the most symbolic foods in traditional Chinese festivals, is integral to both Chinese traditional culture and daily meals. Traditionally, the leaves of different plant species have been used to wrap *zongzi*. The variety of *zongzi* leaves (ZLs) can contribute to the *zongzi*-based cultural diversity. Given the cultural and dietary significance of *zongzi*, the ethnobotanical surveys were carried out, aiming to investigate the diversity of plant species and associated traditional botanical knowledge of ZLs, which could attract particular attention for their further studies.

**Method:**

Both literature studies and field surveys were conducted in the study. The field investigations were carried out from May 2006 to June 2018 throughout China. Ethnobotanical information about ZLs was obtained by direct observation, semi-structured interviews, and key informant interviews.

**Results:**

In total, ZLs from 57 plant species were identified and recorded, belonging to 38 genera and 18 families. Several folk legends have been formed to explain the origin of using plant leaves to pack *zongzi*. Over time, Chinese people have developed diverse traditional botanical knowledge surrounding ZLs, especially regarding the *zongzi* flavor, antiseptic functions, and medicinal values. Based on the literature review, some species of ZLs such as the leaves of *Corchorus capsularis* and *Vernicia fordii* may even pose a potential threat to human health. Presently, in some regions of China, the traditional ZLs, such as *Cocos nucifera*, *Tilia tuan*, and *Zizania latifolia*, are being substituted by commercialized ZLs such as *Phragmites australis* and *Indocalamus tessellatus*.

**Conclusion:**

A variety of traditional ZLs have been discovered in China. Although diverse traditional knowledge exists in China surrounding the usage of ZLs, some species may have the potential of threatening human health. Therefore, further explorations are necessary to comprehensively evaluate traditional ZLs, the results of which could help to conserve the cultural diversity of *zongzi*, to guarantee food safety, and to encourage the uses of plant leaves in food, medicine, and environmental management, for our human health.

## Background

The Dragon Boat Festival, one of the most significant traditional festivals in China, has been celebrated for over 2000 years, occurring on the fifth day of the fifth month in the Chinese lunar calendar [1]. It is also named the *Zongzi* Festival, since eating *zongzi* is a widespread custom to celebrate this festival all over China [2]. *Zongzi*, also named *Jiao Shu* and *Tong Zong*, is a traditional Chinese rice-pudding, which is made of glutinous rice stuffed with different fillings, and then wrapped in plant leaves that are used only for wrapping purposes instead of consumption. Additionally, *zongzi* also plays an indispensable role in daily meals in China [2]. Even though *zongzi* has a distinct cultural significance for Chinese people, *zongzi*-like food is prevalent and carries cultural significance in many other countries and regions, such as Japan, Korea, the Philippines, and Latin America. In Japan, the *zongzi*-like food called *Chimaki* is made of rice flour and is also essential to the Dragon Boat Festival, while in Mexico, *tamales* is made of maize-based dough to celebrate Day of the Dead [3, 4]. In Southeast and East Europe, *sarma* or *dolma*, usually made of rice, bulgur, or minced meat and wrapped in plant leaves, are very common. In Turkey and Caucasus, these foods could be served as festivity meals to celebrate some festivals, such as Easter and Christmas Eve [5].

In present-day China, a great variety of *zongzi* has been developed, with different colors, shapes, fillings, and tastes, thus contributing to the diversification of the *zongzi* culture [6]. There are two most common shapes of *zongzi*: triangular-pyramidal and rectangular. According to the flavor, *zongzi* can be roughly divided into three categories: original, salty, and sweet. The original *zongzi* are only made of white glutinous rice without any other salty or sweet ingredients, while the salty and sweet *zongzi* are made of glutinous rice with the addition of other salty or sweet ingredients. The ingredients added to *zongzi* vary from region to region [7]. In addition to meat like pork and chicken, different parts of plants have been developed to be used as seasonings, such as the flowers of *Nelumbo nucifera*, the fruits of *Ziziphus jujuba*, the seeds of *Castanea mollissima*, and the leaves of *Clausena lansium* and *Perilla frutescens* [6].

Regardless of the category of *zongzi*, after all the fillings are completely prepared, *zongzi* is traditionally wrapped by plant leaves of different species called *zongzi* leaves (ZLs) before they are steamed or boiled. The species of ZLs used depends on regional traditions and geographical locations [6, 8]. The ideal ZLs should meet the requirements of non-contamination, integrity, proper size, pleasant fragrance, preferable flexibility, and tolerance to steaming or boiling [8, 9]. These leaves can be collected from the wild and sold on the market immediately. However, commercially, they are usually air-dried for dehydration in long-term storage in order to eliminate the limitation of regionalism and seasonality [10].

In recent years, the development of biodegradable packaging materials for food has received increased attention since petroleum-based plastics have caused serious environmental contamination because of the resistance to degradation [11]. Renewable natural resources can be effective for the development of biodegradable packing materials [12]. Thus, ZLs with packaging functions may provide a new opportunity for the development and utilization of environmentally friendly packaging materials. In addition to the importance of their packaging properties, ZLs can contribute to the flavor and storage time of *zongzi*. It was reported by Maite et al. [4] that the flavor of *tamales*, the *zongzi*-like food from Mexico, was affected by the plant leaves used to wrap them.

Ethnobotanical surveys focusing on the plant leaves used as wrapping materials for food have been reported in some countries and regions [4, 5, 13]. For example, 21 species of plant leaves have been reported to wrap *tamales* in the Mexican state of Veracruz [4], and the leaves from 87 botanical taxa were used to wrap *sarma* in Turkey and the Balkans [5]. In addition, plant leaves used to wrap food were discovered from time to time when researchers conducted ethnobotanical investigations [14–18]. For instance, nine species of plant leaves were used to wrap food like *tamales* which were cooked in earth ovens located in Maya Lowlands [18]. Although a few species of ZLs have been sporadically reported in publications, most of them were published in Chinese [6, 9, 10, 19]. No studies, to the best of our knowledge, have been carried out to investigate the ethnobotanical importance of ZLs in China. In view of the cultural and dietary significance of *zongzi*, the ethnobotanical surveys of ZLs were conducted from May 2006 to June 2018. The aim of the present study was to investigate the plant species and associated traditional knowledge of ZLs, which could help with the conservation of the cultural diversity of *zongzi*, and be of interest to scientific researchers studying the traditional uses of ZLs. If the associated traditional knowledge can be recorded and understood, it would make contributions to food safety and to the further development and utilization of ZLs in the fields of food, medicine, environmental sanitation, and more broadly, for the sake of our human and environmental health.

## Materials and methods

### Literature studies

A large quantity of records about *zongzi* has been discovered in ancient literatures. Collections from the National Library of China, together with books from ancient to recent times were investigated and examined. Information on the plant species recorded in *Flora of China* (English version) has been intensively studied. In addition, information from databases including Web of Science (WoS), Science Direct, Google Scholar, PubMed, and the Chinese databases such as WP (China Science and Technology Journal Database), Wanfang and CNKI (China National Knowledge Infrastructure) were used in the study.

### Field surveys

**Fig. 1:**
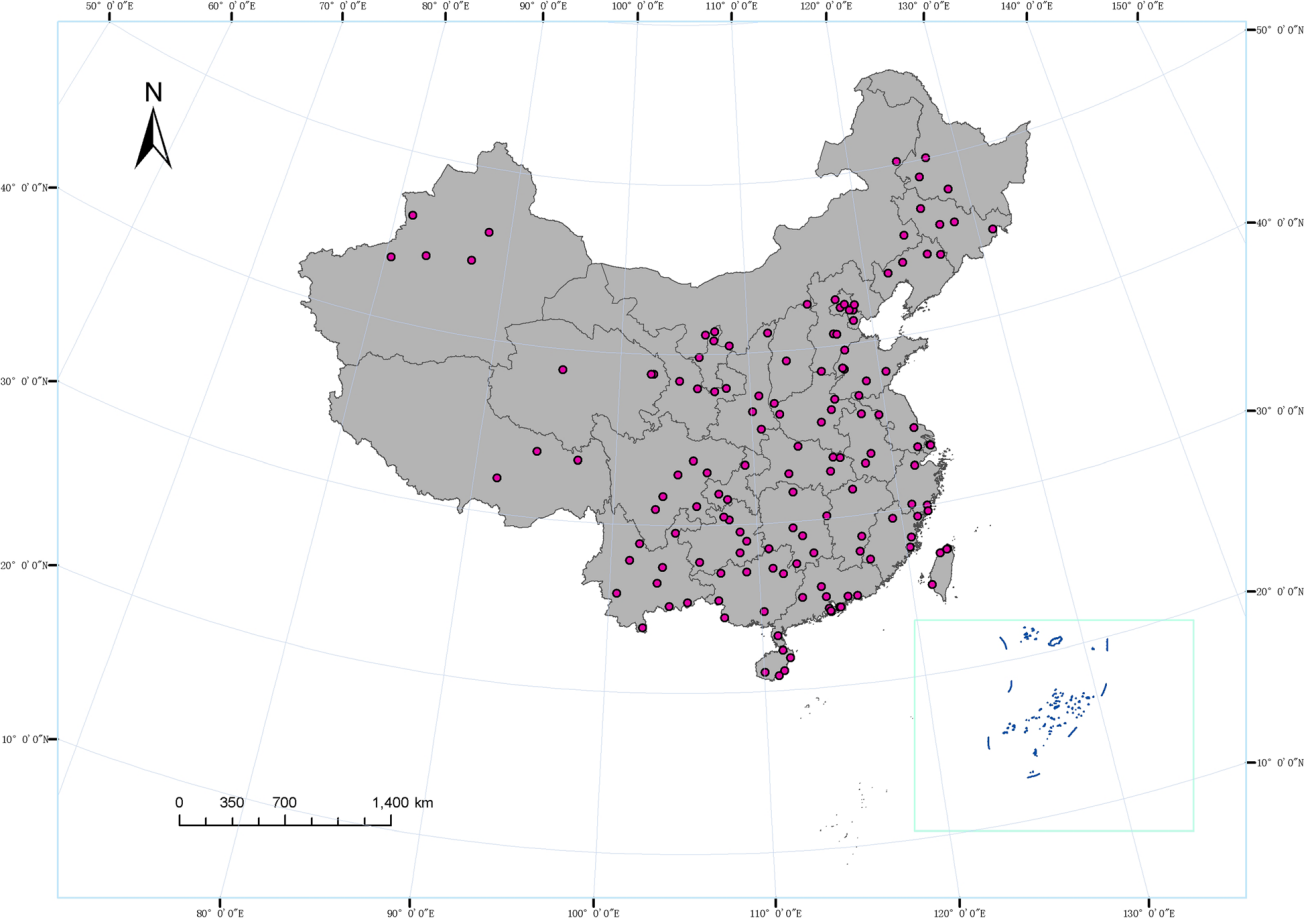
Investigation areas (county or county-level city district) in each province

Our research group has focused on the ZLs for a long time. Here, we recorded and summarized all the ethnobotanical surveys concerning the ZLs by our group. The surveys were mainly conducted close to the Dragon Boat Festival because the *zongzi* were prevalent during this festival, which was beneficial for us to identify the species of ZLs and to investigate the associated traditional knowledge. The ethnobotanical investigations of ZLs were conducted on 31 separate occasions throughout China from May 2006 to June 2018, including 23 provinces, 5 autonomous regions, 4 municipalities, and 2 special administrative regions. Two to nine areas (county or county-level city/district) were investigated in each province. In total, 143 areas throughout China were studied (Fig. 1). In each area, 2 to 5 villages were surveyed and 5–10 people in each village who had traditional knowledge of ZLs were chosen to interview. A total of 3603 informants including 1701 males and 1902 females between 18 and 87 years of age were interviewed. Informants could be characterized as belonging to the following ethnic groups: Mongolian, Tibetan, Uygur, Hui, Miao, Yi, Zhuang, Buyi, Man, Korean, Dong, Bai, Yao, Hani, Tujia, Dai, Li, She, Shui, Qiang, Maonan, Lisu, Jinuo, and Gaoshan ethnic groups, as well as the Han people, who represent the major linguistic group in China.

Several different ethnobotanical methods, including direct observation, semi-structured interviews, and key informant interviews [20] were employed to collect the ethnobotanical data. When conducting surveys, we strictly followed the ethical guidelines issued by the American Anthropological Association (www.aaanet.org) and the International Society of Ethnobiology (http://www.ethnobiology.net). During our surveys, the scientific name, family name, Chinese name, life form, medicinal value, and main distribution of ZLs were recorded. Voucher specimens collected from the various regions were determined and identified by the authors based on *Flora of China* (http://flora.huh.harvard.edu/china), and the nomenclature standards of plant species were referred to *The Plant List* (http://www.theplantlist.org/). The voucher specimens were deposited in the Herbarium of the Minzu University of China.

## Results and discussion

### Diversity of plant leaves used for *zongzi*-wrapping

**Fig. 2:**
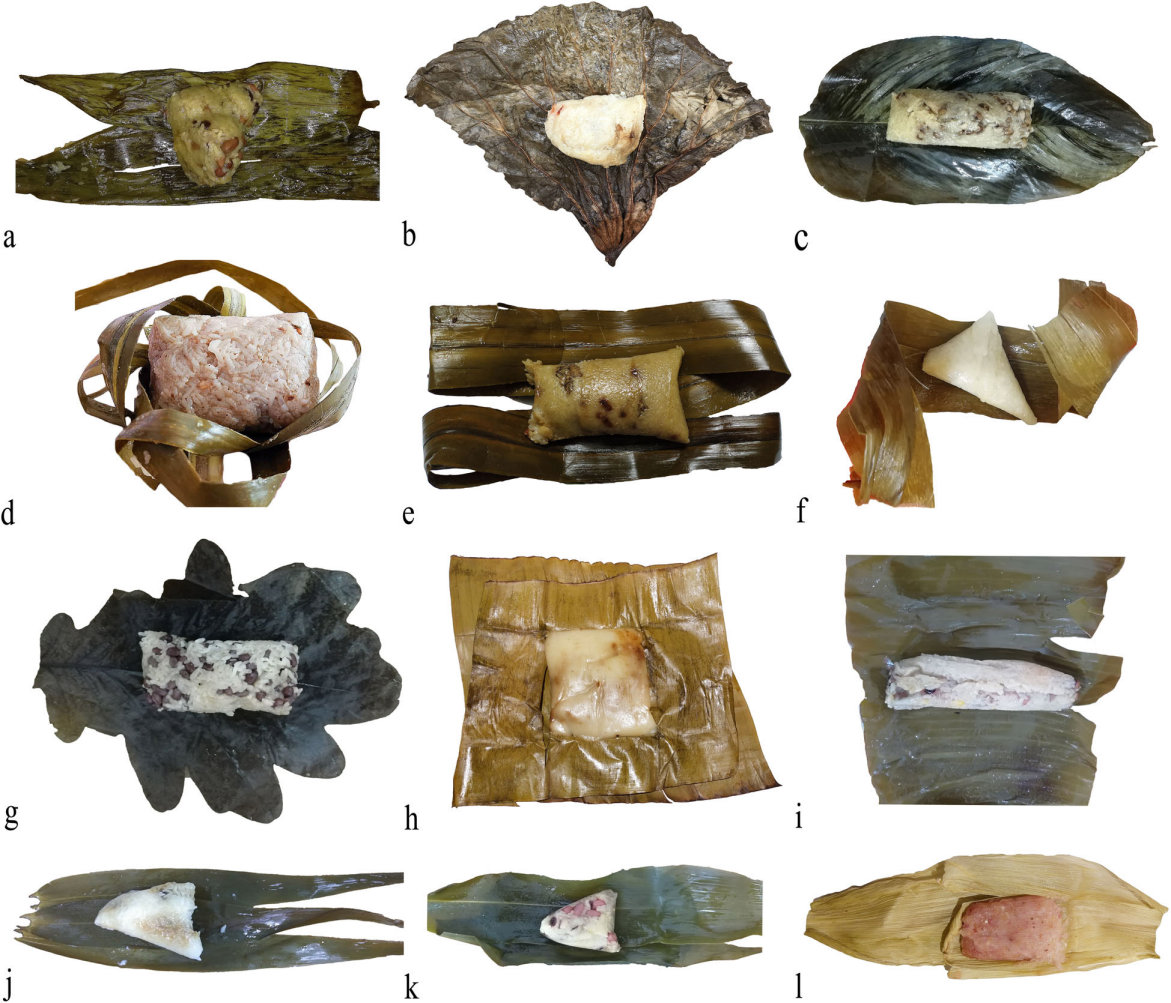
Some species of plantleaves used to wrap *zongzi* (**a**: *Indocalamus tessellatus*; **b**: *Nelumbo nucifera*; **c**: *Phrynium capitatum*; **d**: *Cocos nucifera*; **e**: *Pandanus tectorius*; **f**: *Saccharum officinarum*; **g**: Quercus dentata; **h**: *Musa basjoo*; **i**: *Musa nana*; **j**: Phragmites australis; **k**: Thysanolaena maxima; **l**: *Zea mays*)

**Fig. 3:**
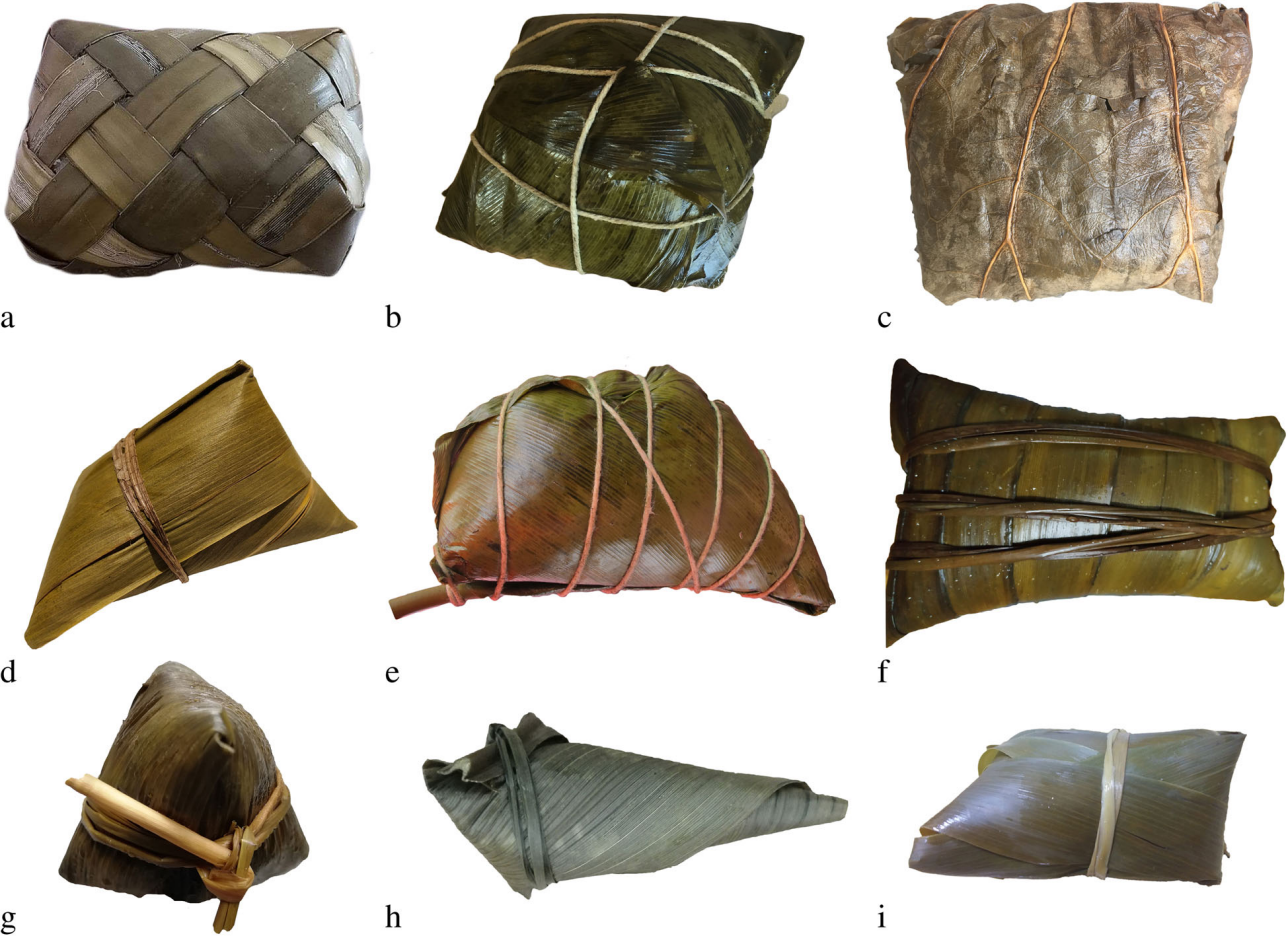
Some shapes of *zongzi* with different species of ZLs (**a**: *Cocos nucifera*; **b**: *Phrynium hainanense*; **c**: *Nelumbo nucifera*; **d**: *Thysanolaena maxima*; **e**: *Phrynium capitatum*; **f**: *Pandanus tectorius*; **g**: *Indocalamus tessellatus*; **h**: Aspidistra sichuanensis; **i**: Phragmites australis)

**Fig. 4:**
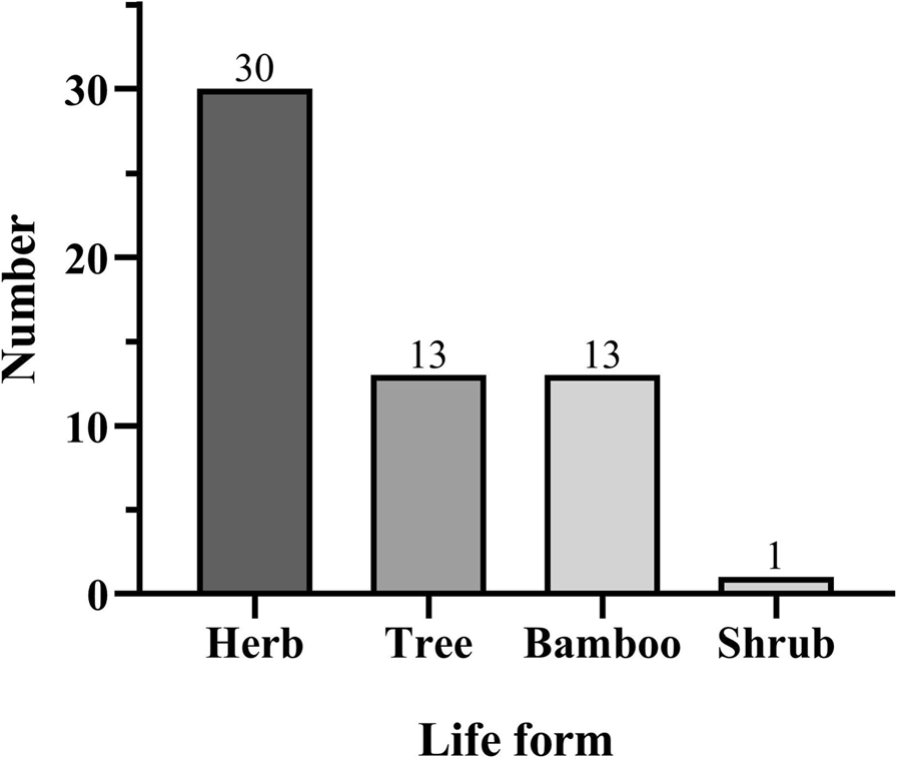
Life form of plant species

China harbors great plant biodiversity, with about 34,000 species of higher plants discovered and recorded [21]. With such diverse plant resources, people in different regions of China have generated different traditions to use local plant species to wrap *zongzi*. On the basis of investigations in China, a total of 57 plant species were documented and identified, falling into 38 genera and 18 families (Table 1). Some examples of *zongzi* with different shapes wrapped by plant leaves are shown in Figs. 2 and 3. There was only one species, namely, *Podocarpus nagi*, that belonged to gymnosperm, while others were all categorized into angiosperm (Table 1). Among the plant families, the most dominant family was Gramineae with 20 species (35.1%), followed by Musaceae (10.5%) Zingiberaceae (8.8%), Liliaceae (7.0%), and Palmae (7.0%), with 6, 5, 4, and 4 species, respectively. As for the plant genera, *Musa*, *Aspidistra*, and *Indocalamus* were the three most common genera, with 6, 4, and 4 species, respectively, followed by *Alpinia*, *Phrynium*, *Phyllostachys*, *Dendrocalamus*, and *Pandanus*. By contrast, other genera contained one species only (Tables 1 and 2). Within these 57 plant species, 30 plant species were herbaceous (52.6%), while 13 were trees or bamboos (22.8%) (Fig. 4). However, only one species, *Rhapis excelsa*, was liana and no shrubs were discovered. When compared with previous investigations [4, 5, 13], the species of plant leaves were quite different from our findings. In Veracruz of Mexica, Marantaceae, Heliconiaceae, and Araliaceae were the three most dominant families whose leaves were used to wrap *tamales*, and only one common species was found, namely, *Zea mays* whose leaves could be used as ZLs by Chinese people [4]. The species of plant leaves as food wrappers for *sarma* in Turkey and the Balkans are all different from ZLs we investigated mostly because the *sarma* leaves could be eaten while the ZLs were only used for wrapping purposes [5, 13]. Our surveys together with the previous studies highlight the significance of ethnobotanical investigations regarding the plant leaves for food wrapping. Extensive investigations are still worthwhile to be conducted in some places, especially in Latin America and Southeast Asia where people consume *zongzi*-like food [6].

**Table 1:** Plant leaves used to wrap *zongzi* in China based on our field work

No.	Scientific name	Family name	Chinese name	Life form	Medicinal value	Main distribution	Voucher number
1	*Alpinia abundiflora* Burtt and R. M. Sm.	Zingiberaceae	草豆蔻	Herb	Treating rheumatism, invigorating spleen and alleviating emesis	Hainan	MUCH-ZLs-057
2	*Alpinia pricei* Hayata	Zingiberaceae	短穗山姜	Herb	——	Taiwan	MUCH-ZLs-046
3	*Alpinia zerumbet* (Pers.) B. L. Burtt and R. M. Sm.	Zingiberaceae	艳山姜	Herb	Treating rheumatism	Taiwan and Fujian	MUCH-ZLs-039
4	*Amomum villosum* Lour.	Zingiberaceae	砂仁	Herb	Treating rheumatism	Guangdong, Guangxi, Yunnan	MUCH-ZLs-010
5	*Arundo donax* L.	Gramineae	芦竹	Bamboo	Clearing heat and diuresis	Yunnan, Guangxi, and Guizhou	MUCH-ZLs-007
6	*Aspidistra elatior* Blume	Liliaceae	蜘蛛抱蛋	Herb	Diminishing inflammation, hemostasis, treating rheumatism and analgesia	Yunnan and Guizhou	MUCH-ZLs-008
7	*Aspidistra oblanceifolia* F. T. Wang et K. Y. Lang	Liliaceae	棕叶草	Herb	——	Yunnan and Guizhou	MUCH-ZLs-044
8	*Aspidistra sichuanensis* K. Y. Lang et Z. Y. Zhu	Liliaceae	四川蜘蛛抱蛋	Herb	——	Sichuan, Yunnan, and Guizhou	MUCH-ZLs-043
9	*Aspidistra zongbayi* K. Y. Lang et Z. Y. Zhu	Liliaceae	粽粑叶	Herb	Clearing heat, detoxification, hemostasis, and diuresis	Yunnan and Guizhou	MUCH-ZLs-009
10	*Cocos nucifera* L.	Palmae	椰子	Tree	Clearing heat	Hainan	MUCH-ZLs-031
11	*Corchorus capsularis* L.	Tiliaceae	黄麻	Herb	Diminishing inflammation, detoxification, hemostasis, and analgesia	Guangxi	MUCH-ZLs-034
12	*Dendrocalamus giganteus* Munro	Gramineae	龙竹	Bamboo	Clearing heat	Yunnan	MUCH-ZLs-011
13	*Dendrocalamus latiflorus* Munro	Gramineae	麻竹	Bamboo	Clearing heat and detoxification	Southern China	MUCH-ZLs-056
14	*Evodia glabrifolia* (Champ.) N. P. Balakr.	Rutaceae	楝叶吴萸	Tree	Diminishing inflammation and analgesia	Guangxi, Hainan and Fujian	MUCH-ZLs-032
15	*Fargesia fractiflexa* T.P. Yi	Gramineae	扫把竹	Bamboo	——	Yunnan	MUCH-ZLs-012
16	*Firmiana platanifolia* (L.f.) Marsili	Labiatae	梧桐	Tree	Clearing heat and detoxification	Guangdong and Hunan	MUCH-ZLs-041
17	*Hedychium coronarium* J. Koenig	Zingiberaceae	姜花	Herb	Treating rheumatism, analgesia and insomnia	Taiwan	MUCH-ZLs-049
18	*Indocalamus guangdongensis* H. R. Zhao and Y. L.Yang	Gramineae	广东箬竹	Bamboo	Clearing heat and detoxification	Guangdong, Guangxi, and Guizhou	MUCH-ZLs-020
19	*Indocalamus herklotsii* McClure	Gramineae	粽巴箬竹	Bamboo	Clearing heat and detoxification	Guangdong, Guangxi and Hunan	MUCH-ZLs-037
20	*Indocalamus latifolius* (Keng) McClure	Gramineae	阔叶箬竹	Bamboo	Clearing heat and detoxification	Southern China	MUCH-ZLs-013
21	*Indocalamus tessellatus* (Munro) Keng f.	Gramineae	箬竹	Bamboo	Clearing heat, detoxification, hemostasis, and diminishing inflammation	Southern China	MUCH-ZLs-006
22	*Livistona chinensis* (Jacq.) R.Br. ex Mart.	Palmae	蒲葵	Tree	Diminishing inflammation and hemostasis	Yunnan and Hainan	MUCH-ZLs-014
23	*Magnolia officinalis* Rehder and E.H.Wilson	Magnoliaceae	凹叶厚朴	Tree	Treating rheumatism, diminishing inflammation and analgesia	Guangxi	MUCH-ZLs-033
24	*Miscanthus floridulus* (Labill.) Warb. ex K. Schum. and Lauterb.	Gramineae	五节芒	Herb	Clearing heat, detoxification, and diuresis	Fujian and Zhejiang	MUCH-ZLs-028
25	*Monocladus amplexicaulis* Chia et al.	Gramineae	芸香竹	Bamboo	Clearing heat and treating rheumatism	Guangxi	MUCH-ZLs-045
26	*Musa acuminata* Colla	Musaceae	小果野蕉	Herb	Clearing heat	Yunnan and Guangxi	MUCH-ZLs-015
27	*Musa balbisiana* Colla	Musaceae	野蕉	Herb	——	Yunnan and Guangxi	MUCH-ZLs-055
28	*Musa basjoo* Siebold and Zucc. ex Iinuma	Musaceae	芭蕉	Herb	Clearing heat and diuresis	Southern China	MUCH-ZLs-001
29	*Musa nana* Lour.	Musaceae	香蕉	Herb	Clearing heat, detoxification, and diuresis	Yunnan, Guangdong, Guangxi and Fujian	MUCH-ZLs-003
30	*Musa sapientum* L.	Musaceae	大蕉	Herb	Clearing heat and diminishing inflammation	Yunnan, Guangdong, and Guangxi	MUCH-ZLs-016
31	*Musa itineras* Tutcher	Musaceae	野芭蕉	Herb	Clearing heat and antimalarial effect	Yunnan, Guizhou, and Guangxi	MUCH-ZLs-002
32	*Nelumbo nucifera* Gaertn.	Nymphaeaceae	莲	Herb	Clearing heat, detoxification, and hemostasis	Jiangsu, Zhejiang, Guangdong, and Hainan	MUCH-ZLs-021
33	*Pandanus austrosinensis* T. L.Wu	Pandanaceae	露兜草	Herb	Clearing heat, detoxification, and diminishing inflammation	Guangdong and Hainan	MUCH-ZLs-022
34	*Pandanus tectorius* Parkinson ex Du Roi	Pandanaceae	露兜树	Tree	Clearing heat and diuresis	Guangdong and Hainan	MUCH-ZLs-023
35	*Perilla frutescens* (L.) Britton	Labiatae	紫苏	Herb	Treating common cold, alleviating emesis, invigorating spleen and stomach	Liaoning	MUCH-ZLs-052
36	*Phragmites australis* (Cav.) Trin. ex Steud.	Gramineae	芦苇	Herb	Clearing heat and detoxification	Northern China	MUCH-ZLs-029
37	*Phrynium capitatum* Willd.	Marantaceae	柊叶	Herb	Clearing heat, detoxification, hemostasis, relieving sore throat, diminishing inflammation and anti-alcoholism	Guangdong, Guangxi, Hainan and Yunnan	MUCH-ZLs-004
38	*Phrynium hainanense* T. L. Wu and S. J. Chen	Marantaceae	海南柊叶	Herb	Clearing heat	Hainan	MUCH-ZLs-047
39	*Phrynium placentarium* (Lour.) Merr.	Marantaceae	尖苞柊叶	Herb	Clearing heat, detoxification, hemostasis, and diuresis	Guangdong, Guangxi, and Yunnan	MUCH-ZLs-017
40	*Phyllostachys bambusoides* Siebold and Zucc.	Gramineae	桂竹	Bamboo	Clearing heat and detoxification	Southern China	MUCH-ZLs-018
41	*Phyllostachys heteroclada* Oliv.	Gramineae	水竹	Bamboo	Clearing heat and detoxification	Yunnan and Guangxi	MUCH-ZLs-024
42	*Phyllostachys heterocycla* (Carrière) Matsum.	Gramineae	毛竹	Bamboo	Clearing heat, detoxification, diminishing inflammation, alleviating emesis, and relieving cough	Sichuan, Zhejiang, and Henan	MUCH-ZLs-026
43	*Piper sarmentosum* Roxb.	Piperaceae	假蒟	Herb	Diminishing inflammation, treating rheumatism, analgesia, and antimalarial effect	Guangdong and Guangxi	MUCH-ZLs-025
44	*Pleioblastus amarus* (Keng) Keng f.	Gramineae	苦竹	Bamboo	Clearing heat, detoxification, and removing the phlegm	Yunnan and Guizhou	MUCH-ZLs-042
45	*Podocarpus nagi* (Thunb.) Pilg.	Podocarpaceae	竹柏	Tree	Hemostasis and treating common cold	Guangdong and Guangxi	MUCH-ZLs-035
46	*Quercus dentata* Thunb.	Fagaceae	槲树	Tree	Clearing heat, hemostasis, diuresis, and treating hemorrhoid	Shanxi, Henan, and Shandong	MUCH-ZLs-053
47	*Rhapis excelsa* (Thunb.) Henry	Palmae	棕竹	Shrub	Treating rheumatism, hemostasis, and alleviating emesis	Guangxi and Yunnan	MUCH-ZLs-019
48	*Saccharum officinarum* L.	Gramineae	甘蔗	Herb	Clearing heat, detoxification, and hypoglycemic effect	Fujian and Guangdong	MUCH-ZLs-040
49	*Sorghum bicolor* (L.) Moench	Gramineae	高粱	Herb	Diminishing inflammation and treating rheumatism	Hunan, Hubei, and Shandong	MUCH-ZLs-030
50	*Sterculia nobilis* Sm.	Sterculiaceae	苹婆	Tree	Treating rheumatism	Guangdong and Guangxi	MUCH-ZLs-027
51	*Terminalia catappa* L.	Myrtiflorae	榄仁树	Tree	Treating rheumatism, detoxification, relieving cough and hypoglycemic effect	Taiwan	MUCH-ZLs-048
52	*Thysanolaena maxima* (Roxb.) Kuntze	Gramineae	粽叶芦	Herb	Detoxification, treating bronchitis, hepatitis, and diarrhea	Guangdong, Guangxi, Guizhou, and Yunnan	MUCH-ZLs-005
53	*Tilia tuan* Szyszył.	Tiliaceae	椴树	Tree	Treating rheumatism and ostalgia	Beijing	MUCH-ZLs-050
54	*Trachycarpus fortunei* (Hook.) H. Wendl.	Palmae	棕榈	Tree	Hemostasis and diuresis	Hunan, Jiangxi, and Sichuan	MUCH-ZLs-036
55	*Vernicia fordii* (Hemsl.) Airy Shaw	Euphorbiaceae	油桐	Tree	Removing the phlegm and promoting digestion and assimilation	Hunan and Sichuan	MUCH-ZLs-038
56	*Zea mays* L.	Gramineae	玉米	Herb	Clearing heat, hypoglycemic effect	Shandong and Northwestern China	MUCH-ZLs-051
57	*Zizania latifolia* (Griseb.) Turcz. ex Stapf	Gramineae	菰	Herb	——	Jiangsu	MUCH-ZLs-054

**Table 2:** Taxonomic diversity of plant species in China

Family	Number of genera	Percentage	Number of species	Percentage
Gramineae	14	36.8	20	35.1
Palmae	4	10.5	4	7.0
Zingiberaceae	3	7.9	5	8.8
Labiatae	2	5.3	2	3.5
Tiliaceae	2	5.3	2	3.5
Liliaceae	1	2.6	4	7.0
Marantaceae	1	2.6	3	5.3
Musaceae	1	2.6	6	10.5
Pandanaceae	1	2.6	2	3.5
Other families	9	23.7	9	15.8
Total	38	100	57	100

The species of ZLs employed in each province of China are listed in Table 3. Some similarities and differences between the species of ZLs in different regions have been observed, which partly reflected the cultural diversity of *zongzi*. The species of ZLs used by the people in the south of China were much more numerous than that in the north of China (Table 3), which may due to the differences between plant resources, people’s experience and observation towards ZLs and traditional culture. The number of ZLs used in Guangxi Province was the maximum with 28 species, followed by Yunnan, Guangdong, Hainan, and Guizhou provinces with 24, 21, 15, and 15 species, respectively. However, the traditional ZLs had not been found in Biru and Leiwuqi counties, and Chengguan District in Tibet because the local people did not have the traditional custom of eating *zongzi*, or they bought the commercialized ZLs such as the leaves of *Indocalamus herklotsii* from adjacent Sichuan Province due to the local limitations of plant resources. Among these ZLs, the leaves of *Indocalamus* spp. were the most common ZLs in Southern China; however, the leaves of *Phragmites australis* were found to be the most dominant in Northern China. Additionally, the husk leaves or leaves of *Z. mays* were regarded as traditional ZLs in 13 provinces located in both the south and north of China, which may partly be because of the widespread cultivation and people’s similar traditional knowledge related to *Z. mays*.

**Table 3:** The species of ZLs used in each province of China

Province	Investigated area (county or county-level zone)	Linguistic group	Species of ZLs (No.)	Number
East China				16
Shandong	Zhuchengshi, Dongchangfu, Pingyi, Decheng	Han	36, 46, 49, 56	4
Jiangsu	Gaoyou, Peixian, Sihong, Wuzhong	Han	32, 36, 57	3
Anhui	Tongqiao, Lujiang, Huaining, Lixin	Han	20, 36	2
Zhejiang	Taishun, Ruian, Jingning, Putuo, Xihu	Han, She	21, 24, 32, 36, 42	5
Fujian	Xiapu, Hanjiang, Shanghang, Cangshan	Han, She	3, 13, 14, 20, 21, 24, 28, 40, 48	9
Shanghai	Hongkou, Pudong, Fengxian	Han	20, 21, 32	3
South China				36
Guangdong	Sanxiang, Qingcheng, Haifeng, Huidong, Deqing, Leizhou, Haizhu	Han, Yao	4, 13, 16, 18, 19, 20, 21, 28, 29, 30, 32, 33, 34, 37, 39, 40, 43, 45, 48, 50, 52	21
Guangxi	Yongfu, Jingxi, Leye, Huanjing, Lingshan, Pingxiang, Pingle	Han, Zhuang, Yao	4, 5, 9, 11, 13, 14, 18, 19, 20, 21, 23, 25, 26, 27, 28, 29, 30, 31, 34, 37, 39, 40, 41, 43, 45, 47, 50, 52	28
Hainan	Ledong, Wenchang, Meilan, Lingshui, Wanning	Han, Li, Miao	1, 10, 14, 20, 21, 22, 27, 28, 29, 32, 33, 34, 37, 38, 40	15
Central China				17
Hubei	Xiangyang, Hongan, Hongshan, Xiaoting	Han	28, 36, 49, 54, 56	5
Hunan	Guiyang, Lixian, Qidong, Xinshao, Jianghua, Tongdao	Han, Dong, Yao	6, 13, 16, 19, 20, 21, 28, 29, 37, 40, 49, 54, 55	13
Henan	Lushi, Xiping, Taikang, Minquan	Han	20, 21, 36, 42, 46, 56	6
Jiangxi	Huichang, Ningdu, Shangli, Dean	Han	13, 20, 21, 28, 36, 54	6
North China				6
Beijing	Yanqing, Haidian, Shunyi	Han, Man	36, 53	2
Tianjin	Baodi, Hedong, Hexi	Han	36	1
Hebei	Cixian, Xianghe, Lixian, Suning	Han	36	1
Shanxi	Liaocheng, Ruicheng, Xiaoyi, Yanggao	Han	36, 46	2
Inner Mongolia	Keerqin, Alashanzuoqi, Zhalantun, Etuokeqianqi	Han, Mongolian	36, 56	2
Northwest China				6
Ningxia	Jingyuan, Dawukou, Xingqing, Shapotou	Han, Hui	36	1
Xinjiang	Akesu, Tianshan, Cabuchaerxibo, Kuche, Bohu	Han, Uighur	36, 56	2
Qinhai	Chengxi, Huangzhong, Geermu	Han, Tibetan	36, 56	2
Shanxi	Luonan, Baqiao, Baishui, Shenmu	Han	32, 36, 46, 56	4
Gansu	Anning, Zhenyuan, Huining	Han	28, 32, 36, 56	4
Southwest China				30
Sichuan	Nanxi, Pengxi, Hanyuan, Fucheng, Mianning, Chongzhou	Han, Yi, Qiang	6, 8, 13, 20, 21, 28, 36, 40, 42, 54, 55, 56	12
Yunnan	Panlong, Ludian, Eshan, Mengla, Maguan, Shuangjiang, Jinping, Xiangyun, Yongsheng	Han, Dai, Hani, Jinuo, Yi, Yao, Lisu, Tujia, Maonan	4, 5, 6, 7, 8, 9, 12, 13, 15, 20, 21, 22, 26, 27, 28, 29, 30, 37, 39, 40, 41, 44, 47, 52, 56	24
Guizhou	Yuqing, Suiyang, Tongzi, Xingren, Sandu, Taijiang	Han, Miao, Dong, Buyi, Shui	5, 6, 7, 8, 9, 13, 18, 20, 21, 28, 31, 32, 40, 44, 52	15
Tibet	Chengguan, Leiwuqi, Biru	Tibetan	——	0
Chongqing	Nanchuan, Jiulongpo, Kaizhou	Han	20, 21, 28, 56	4
Northeast China				3
Jilin	Fengman, Nanguan, Yanji, Qianan	Han, Korean	36, 56	2
Heilongjiang	Yian, Daowai, Duerbote	Han, Man, Korean	36, 56	2
Liaoning	Shuangta, Yinchuan, Qingyuan, Fuxin	Han, Korean, Mongolian	35, 36	2
Other areas				6
Taiwan	Xinzhu, Annan, Wenshan, Banqiao	Han, Gaoshan	2, 3, 17, 21, 51	5
Xianggang	Quanwan, Shatian	Han	20, 21, 28	3
Macao	Dangzai, Datang	Han	20, 21	2

The numbers of species of ZLs are equivalent to the numbers in Table 1

According to our surveys, some similarities and differences of ZLs among or within ethnic groups were discovered. The ZLs of *Phrynium capitatum* were widely used by the Han people in the west and south of Guangdong Province. However, it was hardly found among the Han people in the east, mainly due to the distribution area of *P*. *capitatum*. It was commonly believed by the Han people in Shanghai that the usage of the traditional ZLs of *Indocalamus* spp. was originated from the Han communities in Anhui Province because of the cultural communication in the course of economic exchange. Even though *Piper sarmentosum* is widely distributed in Guangxi Province, it was told that *P*. *sarmentosum* leaves were mainly used by the Zhuang people in East Guangxi. It was rarely used by the same ethnic group in other parts of Guangxi because, for the Zhuang people in the east part, the knowledge that *P*. *sarmentosum* leaves could be used as ZLs was inherited from their ancestors for a long time. Therefore, the similarities and differences of the species of ZLs among and within ethnic groups could be considered the results of plant distribution, the heritage of traditional knowledge, and cultural exchange.

The leaves of *Zizania latifolia* represent the earliest ZLs, which have been used since the Spring and Autumn period of China (770–476 BC) [22]. Even though using the ZLs of *Z*. *latifolia* was prevalent in ancient times, it has now greatly lost its popularity based on our field investigations. According to the areas we investigated, only people in Suzhou City of Jiangsu Province in China still used this species as one of the traditional ZLs. The leaf of *Melia azedarach* was another ancient ZL, which was recorded in the Chinese ancient book, *Xu Qi Xie Zhi*, written by Wu Jing of Southern Dynasties of China (420–589 AD) [23]. However, it had not been discovered as being used by people to wrap *zongzi*. Studying the inheritance and change of traditional ZLs in each area could prove significant to the conservation of biodiversity of ZLs.

In addition to plant leaves, the shells of bamboo shoots, according to our interviews, can also be traditionally used to wrap *zongzi* in some regions such as Sichuan, Hunan, and Zhejiang provinces. Some species of plant leaves with correct length and good flexibility are good resources to bind *zongzi* apart from their wrapping abilities, such as the leaves of *Phragmites australis*, *Cocos nucifera*, and *Livistona chinensis*. Traditionally, the leaves or stems of *Imperata cylindrica*, *Iris tectorum*, *Oryza sativa*, *Trachycarpus fortune*, *Typha angustifolia*, and *Cyperus malaccensis* can be used as binding materials as well. However, it has become more and more common to use strings made of cotton or flax to bind *zongzi* at present because of their convenience and low prices.

### The folk legends of ZLs

According to our surveys, some folk legends of ZLs had been widely spread. It is generally believed that both the Dragon Boat Festival and the traditional custom of eating *zongzi* are to commemorate the great Chinese patriotic poet, Qu Yuan (339–278 BC), who drowned himself to death for his country in the Miluo River (Yueyang City, Hunan Province, at present). At that time, when people heard the news of his suicide, they fell into a deep sadness. Then, it was agreed that scattering the cooked glutinous rice into the river as a sacrifice for Qu Yuan was beneficial to express these feelings. However, the food thrown into the river was mainly robbed and eaten by Jiaolong, the mythical dragon-like creature at the time. Fortunately, Jiaolong could be effectively deterred by the plant leaves of *Melia azedarach*. Using this knowledge, the people started using plant leaves to wrap glutinous rice before it was thrown into the river so as to protect the food for Qu Yuan from being eaten by Jiaolong. This folk legend was widely spread and passed down from generation to generation throughout China. Over time, different ZLs from various plant species are gradually adopted on the Dragon Boat Festival in China. Our investigations were consistent with those recorded in the ancient books, *Xu Qi Xie Zhi* and *Jing Chu Sui Shi Ji* [23], which, to some extent, indicated the stability of cultural inheritance of ZLs in China.

Interestingly, a particular folk legend concerning the leaves of *Miscanthus floridulus* was well known by the She people in Chibi Village of Fujian Province in Southern China. This legend is related to the revenge and atrocity of Zhu Yuanzhang (1328–1398 AD), who was the first emperor of the Ming Dynasty (1368–1644 AD) [24]. When Zhu Yuanzhang was a cowboy in his childhood, he did not take care of his cow. As a result, his cow usually audaciously trampled and ate the vegetables cultivated by the She people. One time, when an old She woman found that the cow was eating her vegetables, she was so angry that she expelled it with a whip. After knowing that his cow was seriously lashed, Zhu Yuanzhang became furious and made a promise to revenge one day. When he became the emperor of the Ming Dynasty, he still remembered the humiliation that he experienced in his childhood and he commanded his army to kill the She people. Therefore, the She people had to leave their hometowns, those of whom in Guangdong Province escaped to the remote mountains distributed in the east of Fujian Province. They were so afraid of being killed that they could not collect the bamboo leaves that grew down the hill at lower altitudes, which they originally used to wrap *zongzi* during the Dragon Boat Festival. One patriarch came up with a good idea that the leaves of *M*. *floridulus* could also be used as ZLs. This novel way to wrap *zongzi* was widely spread and accepted by the She people. From then on, the tradition of using the ZLs of *M*. *floridulus* had been gradually formed. While the origin and authenticity of this legend remain to be determined and validated, it is possible that these legends about ZLs could be partly responsible for the diversity of the traditional culture of the Dragon Boat Festival.

### Collection and processing of ZLs

According to our interviews, despite a diversity of plant leaves used, the people across different regions had almost the same processes to collect and process ZLs. In general, the fresh healthy ZLs are collected during the Dragon Boat Festival in the mountains or home gardens and then washed with fresh water to remove dirt and dust. After the leaves are cleaned up, they are immersed in boiling water until they become soft and flexible enough to wrap the prepared glutinous rice. The necessity of preliminary heat treatment was also reported by Dogan and colleagues [5] when the leaves were used to wrap *sarma* in Turkey or the Balkans. The leaves used for *sarma* are eaten afterward, but ZLs are just for the package. Once packed with ZLs and bound tightly, *zongzi* as a whole is then boiled or steamed until they were suitable to eat. The similarity in the collection and processing of ZLs exemplifies the unity of culture surrounding the *zongzi* or the Dragon Boat Festival in China.

### Contribution to *zongzi* flavor

The *zongzi* flavor can be affected by both the inside fillings and the ZLs [9]. It was reported that some species of plant leaves such as *Oreopanax flaccidus* leaves could add a unique flavor to *tamales* [4]. According to our surveys, in general, over 80% of people preferred to use the ZLs which they believed were more fragrant. It was commonly believed that ZLs could vastly contribute to the *zongzi* flavor. For example, people in some regions of Southern China have found that *zongzi* wrapped by the leaves of *Nelumbo nucifera*, *Piper sarmentosum*, *Indocalamus* spp., and *Musa* spp. have a special fragrance, and it was believed by the Li ethnic group in Hainan Province that *zongzi* with *Cocos nucifera* leaves had coconut-like flavor that came from the leaves. The areas where people are familiar with the flavor contributions of ZLs were provided in Additional file 1: Table S1.

The flavor compounds from *Alpinia zerumbet* [25], *Hedychium coronarium* [26], *Indocalamus latifolius* [27], *I*. *tessellatus* [28], *Musa acuminate* [29], *Nelumbo nucifera* [30], *Perilla frutescens* [31], *Quercus dentata* [32], and *Terminalia catappa* [33] have been previously characterized and identified. For instance, nine critical flavor components have been identified by GC-MS from the leaves of *I*. *tessellatus* including *p*-vinylphenol, *p*-vinylguaiacol, diphenylmethanone, 2, 2′-diethylbiphenyl, 2, 6-diisopropylnaphthalene, (*Z*)-phytol, eicosanenitrile, 2-phenyltridecane, and (*E*)-phytol [28]. These results therefore supported the traditional knowledge that these species of ZLs could contribute the *zongzi* flavor. However, as far as we know, there have been no reports on the flavor compounds of other ZLs yet. Consequently, further studies are greatly needed to fully characterize the aroma-active constituents, which will encourage them to be developed as naturally refreshing agents for our environmental sanitary or natural flavor substances with health-promoting properties for food.

### Antiseptic functions of ZLs

According to our interviews, apart from the contribution to the *zongzi* flavor, some species of ZLs are believed to have antiseptic properties, which results in relatively long-term storage time of *zongzi* under natural conditions. The Dragon Boat Festival is celebrated at the end of the spring and the beginning of the summer when the food is easily attacked by spoilage organisms because of the suitable temperature and humidity [34]. Thus, ZLs with antiseptic functions are more favorable. According to our surveys in the local areas, 26 species of ZLs were considered to be responsible for the shelf life of *zongzi*, including the leaves of *Aspidistra* spp., *Cocos nucifera*, *Evodia glabrifolia*, *Fargesia fractiflexa*, *Indocalamus* spp., *Magnolia officinalis*, *Miscanthus floridulus*, *Monocladus amplexicaulis*, *Musa* spp., *Pandanus tectorius*, *Phragmites australis*, *Phrynium capitatum*, *Piper sarmentosum*, *Quercus dentata*, and *Thysanolaena maxima*. For instance, according to the interviews, the sweet *zongzi* with ZLs of *I*. *latifolius*, *P*. *australis*, or *Q*. *dentata* would not be spoiled during 10 days of storage in summer, and the salty-meat *zongzi* with ZLs of *P*. *tectorius*, *M*. *basjoo*, or *C*. *nucifera* could still stay fresh within five days under natural ventilation condition. The areas where people are familiar with the antiseptic functions of ZLs were listed in Additional file 1: Table S1.

At present, antimicrobial properties of packaging materials have attracted public concerns and have been included in the next generation of food packaging [35]. Based on previous studies, the polar extracts or the essential oils from the leaves of *A*. *elatior* [36], *C*. *nucifera* [37], *I*. *latifolius* [38], *I*. *tessellatus* [39], *M. officinalis* [40], *M*. *acuminata* [41], *M*. *sapientum* [42], *P*. *tectorius* [43], *P*. *australis* [44], *P*. *capitatum* [19], *P*. *sarmentosum* [45, 46], *Q*. *dentata* [47], and *T*. *maxima* [19] showed good antimicrobial activities. For instance, the essential oils of the leaves of *P*. *capitatum* and *T*. *maxima* presented considerable activity against spoilage organisms such as *Aspergillus fumigatus* and *Candida albicans*, with MIC (minimum inhibitory concentration) ranging from 64 to 1024 mg/mL [19], and the acetone extracts from the leaves of *M*. *acuminata* showed significant antifungal activities against *Aspergillus terreus* and *Penicillium solitum* after 5 days, with the inhibition rate of mycelial growth of 81.1 and 45.6%, respectively [41]. These results are in accordance with the traditional knowledge that these traditional ZLs have antiseptic functions, suggesting the potential utility of associated traditional botanical knowledge surrounding ZLs.

Previous research showed that the fruits extracts containing flavonoids from *M. balbisiana* exhibited antibacterial activity against *Shigella dysenteriae* ATCC 13313, with MIC value ranging from 5 to 10% w/v [48], and the extracts from rhizomes and flowers of *M*. *basjoo* showed antimicrobial activity against *Staphylococcus aureus* and methicillin-resistant *Staphylococcus aureus* [49]. However, to the best of our knowledge, the anti-microbial activities of the leaves of both *M. balbisiana* and *M*. *basjoo* have not been reported yet. Furthermore, no studies have been yet reported regarding the antimicrobial properties of ZLs of *A*. *oblanceifolia*, *A*. *sichuanensis*, *A. zongbayi*, *E*. *glabrifolia*, *F. fractiflexa*, *I*. *guangdongensis*, *I*. *herklotsii, M. amplexicaulis*, *M*. *floridulus*, *M*. *nana*, *Musa itineras*, *P*. *hainanense*, and *P*. *placentarium*, even though people believe that they can increase the storage time of *zongzi*.. Therefore, further studies on the antimicrobial bioactivity of ZLs are worthwhile to be conducted. The ZLs with antiseptic functions are expected to be further developed as biodegradable packaging materials for food, and the bioactive compounds have great potential to be applied in food preservation and drug development.

### Medicinal value of ZLs

**Fig. 5:**
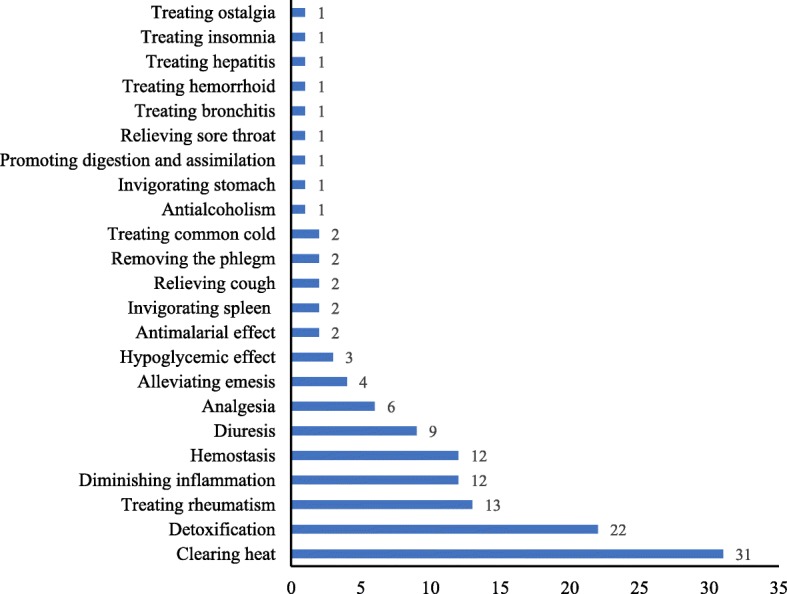
Medicinal values of ZLs

According to our survey, among the 57 species of ZLs, 51 were known to have medicinal values, accounting for 89.5%, some of which had multiple medicinal effects, such as the leaves of *Alpinia abundiflora*, *Aspidistra zongbayi*, *Indocalamus tessellatus Phrynium capitatum*, and *Piper sarmentosum* (Table 1). In total, 23 types of medicinal values were found (Fig. 5). *Heat*-clearing, detoxification, and rheumatism treatment were the three most dominant medicinal functions, with 31 (54.4%), 22 (38.6%), and 13 (22.8%) species of ZLs, respectively, followed by inflammation-diminishing, hemostasis, and diuresis, with 12, 12, and 9 species of ZLs, respectively. *Heat*-clearing is a concept in traditional Chinese medicine (TCM). In TCM, *heat* is one of the main pathogenic factors which could cause disturbance in human body, such as oral ulcer and urethritis [50, 51]. Besides, there were 6, 4, and 3 species of ZLs used to relieve pain, alleviate emesis and reduce hyperglycemia, respectively. The areas where people characterized the medicinal values of ZLs were listed in Additional file 1: Table S1.

Based on our analysis, 28 of 31 species of ZLs with *heat*-clearing function, 20 of 22 with detoxifying effect, and 10 of 13 with rheumatism-treating value are widely used as traditional ZLs in regions located in Southern China (Tables 1 and 3). The Dragon Boat Festival is celebrated when the weather is hot and humid especially in the south of China. The climatic environment with high humidity and temperature could contribute to the extensive production of internal *heat* (in TCM) in our body, and functional deficiency of liver and kidney [52]. Thus, *zongzi* wrapped by the plant leaves with medicinal functions of *heat* elimination, detoxification, and rheumatism treatment may help people compromise internal *heat* and *wetness*, and then prevent related ailments.

Importantly, among the nine species of ZLs which were useful for diuresis, all species also had a *heat*-clearing function; within 22 with detoxifying function, 19 possessed a medicinal value of clearing *heat*. In addition, five species were also involved in the inflammation-diminishing effect among six species with analgesia value. Further studies should be conducted to clarify the relationship, which will be beneficial to the theoretical constructions for TCM [34].

Nowadays, diabetes mellitus, a common metabolic and endocrine disorder, has posed a great threat to human health globally [53]. Insulin and some synthetic diabetic agents which have certain side effects are still the main drugs used for diabetic therapy clinically [54]. Searching for natural and effective compounds to treat diabetes is still greatly needed [55]. It is worthwhile to mention that people believe the leaves of *Saccharum officinarum*, *Terminalia catappa*, and *Zea mays* have hypoglycemic effects. It was reported that the polysaccharides from the leaves of *S*. *officinarum* and aqueous extracts of *T. catappa* leaves had significant hypoglycemic activities on mice [56, 57], which supported the medicinal value of traditional knowledge. Even though the polysaccharides from corn silk and the phenolic extract of corn seeds were found to have bioactivity for hypoglycemia management [58–60], the hypoglycemic effects of corn leaves have not been reported yet*.* Thus, further studies should be conducted to evaluate the hypoglycemic properties of corn leaves.

Malaria caused by *Plasmodium* parasites, such as *P*. *falciparum* and *P*. *vivax*, is one of the most serious diseases in the world [61]. Although combination therapies with Artemisinin-based drugs are effective to remedy malaria globally, parasites with artemisinin resistance have been emerged in Africa [62, 63]. Hence, continuing investments in the development of new medicines remain urgent [64]. According to our surveys, the ZLs of *Musa wilsonii* and *Piper sarmentosum* possessed antimalarial activities. It is reported by Rahman et al. [65] that methanol and chloroform extracts from *P*. *sarmentosum* showed significant antimalarial effects, supporting our investigated plant knowledge. However, to date, the antimalarial activity as well as the chemical constituents of the leaves of *M*. *wilsonii* have not been clarified. Future research deserves to be conducted to determine its potential ability against malaria.

### Other functions of ZLs

In addition to the functions of flavor contribution, antiseptic activity and medicinal effects, some species of ZLs also have other functions. On the basis of our surveys, the edible value of the leaves of *Piper sarmentosum* has been recognized by local people in Southern China, especially in Guangdong and Guangxi provinces. The leaves from this species could serve as delicious seasonings that could be added into dishes, such as soup, river-snail cuisine, and beef patty. In Thailand, the leaves of *P*. *sarmentosum* are traditionally used as food as well [66]. In Lingshui Li Autonomous County of Hainan Province, the leaves of *Pandanus austrosinensis* not only could be used to wrap *zongzi*, but also could be used for the weaving of straw mats, hats and baskets, the constructions of house barriers, and even the mythical functions of frightening and expelling the evils. Interestingly, it was told by the local people in Shagang Village that the leaves of *P*. *austrosinensis* could be used as fences to prevent intruders because of its spiny leaves. Consequently, the leaves of *P*. *austrosinensis* provide convenience for people both materially and mentally*.* People in Cimuchuan Village (Dazhuangke Township, Yanqing County, Beijing) had found that the leaves of *Tilia tuan* could make more contribution to both the sweet taste and spongy texture of *zongzi* when compared with the leaves of *Phragmites australis*.

### Effects of ZLs on food safety

According to our interviews, people generally believe that traditional ZLs are environmental-friendly, non-toxic, and good for human health. However, based on the literature studies [67–70], the ZLs of *Corchorus capsularis* and *Vernicia fordii* may have potential health risks.

The ZLs of *C*. *capsularis* are prevalent in the northern part of Guangxi Province such as Yongfu County. Although the current research showed that the plant species of *C. capsularis* possessed various pharmacological effects such as antioxidant, anti-inflammatory and antipyretic activities, it is considered a toxic plant due to the presence of cardioactive constituents contained, such as Corchoroside A and B in the leaves and seeds [67]. It was reported that Corchoroside A and B from leaf extracts exhibited toxicity to cats with a lethal dose of 0.053–0.0768 and 0.059–0.1413 mg/kg, respectively [68]. In addition, after dietary exposure to *C*. *capsularis* leaves, cattles can suffer from the functional depression of respiratory and vasomotor center, and even death [69]. We should be cautious when using the leaves of *C. capsularis* as packaging materials, and the accessible frequency of *zongzi* with *C. capsularis* leaves should be limited until we better understand its potential toxicity to humankind.

It is prevalent to use the leaves of *V*. *fordii* to wrap *zongzi* in Hunan and Sichuan provinces in China. Even though the roots, leaves, and fruits of *V*. *fordii* have been traditionally used to remedy ailments including sore throats, respiratory illnesses, constipation, and diuresis in East Asian folk medicine [71, 72], it was reported that the whole plant of *V*. *fordii* had some toxicity especially its seeds [70]. Consequently, the potential effects of the leaves of *V*. *fordii* on our human health should not be ignored.

Despite the fact that these two species of ZLs we found may have a potential threat to people’s health, no acute poisoning events have been reported in folk history. Herein, we speculate that the toxic constituents may be destroyed or transferred to non-toxic components by steaming or boiling with high temperature, or the contents of poison-active compounds are not enough to cause acute intoxication. Nevertheless, despite the long history usage of traditional ZLs, studies concerning their effects on human health are greatly lacking. Further studies are urgently necessary in order to guarantee food security for the sake of our human health. The future investigations can focus on toxicological assessments of ZLs, such as acute and subacute tests, and the possibility of detoxification under *zongzi*-making processes.

### Potential impact of commercialization on traditional ZLs

Nowadays, 12 species of ZLs have been commercialized and are easily accessed via online shopping stores: the leaves of *Alpinia zerumbet*, *Indocalamus tessellatus*, *Musa basjoo*, *Musa nana*, *Nelumbo nucifera*, *Phragmites australis*, *Phrynium capitatum*, *Piper sarmentosum*, *Quercus dentata*, *Sterculia nobilis*, *Vernicia fordii*, and *Zea mays*. According to our interviews, some species of traditional ZLs including *Cocos nucifera* used in Wenchang City of Hainan Province, *Tilia tuan* used in Yanqing County of Beijing, and *Zizania latifolia* used in Suzhou City of Jiangsu Province, had been threatened by commercialized ZLs such as the leaves of *I*. *tessellatus* and *P*. *australis*, because of their low price, good quality, and easily accessible advantages. The indivisible interconnections have been recognized between biological and cultural diversity, and the destruction of biodiversity can lead to the loss of associated culture [73]. Thus, once the traditional ZLs used in extremely limited regions, such as the threatened ZLs of *T*. *tuan* and *Z*. *latifolia*, which, according to our surveys, are only utilized by people in Beijing and Suzhou City, respectively, have been substituted by other common leaves, they are likely to be lost irreversibly, along with related culture including associated traditional knowledge. The effects of e-commerce and commercialized ZLs on traditional ZLs should not be neglected. The variety of traditional ZLs in China may decrease, which could threaten the cultural diversity of *zongzi* or the Dragon Boat Festival to some extent. However, the specific influence of commercialized ZLs still needs to be further determined.

## Conclusion

The plant species used for wrapping *zongzi*, the traditional food for celebrating the Dragon Boat Festival in China, depending on regional traditions and local plant species. Using various species of ZLs reflects the fact that Chinese people make good use of the local materials in their regions. A total of 57 plant species (38 genera and 18 families) were documented and identified, among which the leaves of *Indocalamus* spp. and *Phragmites australis* are the most dominant in Southern and Northern China, respectively*.* There are some widespread folk legends about ZLs, which culturally reveal the origins of using plant leaves to wrap *zongzi*. With the traditional uses of ZLs, Chinese people have achieved a wealth of traditional botanical knowledge, particularly in flavor contribution, antiseptic functions, and medicinal effects, some of which are supported by current scientific research. However, some species of traditional ZLs such as the leaves of *C*. *capsularis* and *V*. *fordii* may pose a potential threat to human health. Further studies remain to be conducted to comprehensively evaluate these traditional uses. At present, in some areas, there is a potential possibility that some traditional ZLs, including the ZLs of *Cocos nucifera* and *Tilia tuan*, as well as the most ancient ZLs of *Zizania latifolia*, are threatened and could be replaced by the commercialized ZLs, such as the leaves of *Indocalamus tessellatus* and *Phragmites australis*. However, the potential impact of commercialization on traditional ZLs still needs to be studied. Our study highlights the ethnobotanical knowledge surrounding ZLs, and could provide important clues for their further studies.

## Supplementary information

**Additional file 1: Table S1.** The investigation areas (county level) where people are familiar with the functions of ZLs.

## Data Availability

All data generated or analyzed during this study are included in this published article and its supplementary information files.
