# Supplementary material for: Plant leaves for wrapping zongzi in China: an ethnobotanical study
Source: J Ethnobiol Ethnomed. 2019 Dec 11;15:63. doi: 10.1186/s13002-019-0339-7 (PMC6907129; doi:10.1186/s13002-019-0339-7)
Supplement: Supplementary file 1 — Additional file 1: Table S1. The investigation areas (county level) where people are familiar with the functions of ZLs. [file 13002_2019_339_MOESM1_ESM.docx]

Additional file 1: The investigation areas (county level) where people are familiar with the functions of ZLs

| No. | Plant species | Family name | Area 1 | Area 2 | Area 3 |
| --- | --- | --- | --- | --- | --- |
| 1 | [*Alpinia abundiflora* Burtt & R. M. Sm.](http://www.theplantlist.org/tpl1.1/record/kew-218630) | Zingiberaceae | Wanning (Hainan) | —— | Wanning (Hainan) |
| 2 | [*Alpinia pricei* Hayata](http://www.theplantlist.org/tpl1.1/record/kew-218983) | Zingiberaceae | Wenshan (Taiwan) | —— | —— |
| 3 | *Alpinia zerumbet* (Pers.) B. L. Burtt & R. M. Sm. | Zingiberaceae | Annan (Taiwan); Shanghang (Fujian) | —— | Annan (Taiwan); Shanghang (Fujian) |
| 4 | [*Amomum villosum* Lour.](http://www.theplantlist.org/tpl1.1/record/kew-219541) | Zingiberaceae | Leizhou (Guangdong); Mengla (Yunnan) | —— | Mengla (Yunnan) |
| 5 | [*Arundo donax* L.](http://www.theplantlist.org/tpl1.1/record/kew-396629) | Gramineae | Yongfu (Guangxi); Xingren (Guizhou); Mengla (Yunnan) | —— | Yongfu (Guangxi); Mengla (Yunnan) |
| 6 | [*Aspidistra elatior*Blume](http://www.theplantlist.org/tpl1.1/record/kew-275646) | Liliaceae | Panlong, Ludian (Yunnan); Xinshao (Hunan); Hanyuan (Sichuan) | Panlong (Yunnan); Hanyuan (Sichuan); Suiyang (Guizhou) | Panlong (Yunnan); Xinshao (Hunan); Suiyang (Guizhou) |
| 7 | [*Aspidistra oblanceifolia*F. T. Wang et K. Y. Lang](http://www.theplantlist.org/tpl1.1/record/kew-275646) | Liliaceae | Mengla (Yunnan); Sandu (Guizhou) | Mengla (Yunnan) | —— |
| 8 | [*Aspidistra sichuanensis* K. Y. Lang et Z. Y. Zhu](http://www.theplantlist.org/tpl1.1/record/kew-275646) | Liliaceae | Hanyuan (Sichuan) | Hanyuan (Sichuan) | —— |
| 9 | [*Aspidistra zongbayi* K.](http://www.theplantlist.org/tpl1.1/record/kew-275646) Y. Lang et Z. Y. Zhu | Liliaceae | Ludian, Jinping (Yunnan) | Ludian (Yunnan) | Ludian, Jinping (Yunnan) |
| 10 | [*Cocos nucifera* L.](http://www.theplantlist.org/tpl1.1/record/kew-44645) | Palmae | Lingshui, Ledong (Hainan) | Wenchang, Lingshui, Ledong (Hainan) | Lingshui, Ledong (Hainan) |
| 11 | *Corchorus capsularis*L. | Tiliaceae | Yongfu (Guangxi) | —— | Yongfu (Guangxi) |
| 12 | Dendrocalamus *giganteus* Munro | Gramineae | Xiangyun, Shuangjiang (Yunnan) | —— | Xiangyun (Yunnan) |
| 13 | *Dendrocalamus latiflorus* Munro | Gramineae | Haifeng (Guangdong); Maguan (Yunnan); Xingren (Guizhou); Mianning (Sichuan) | —— | Huidong, Haifeng (Guangdong); Mianning (Sichuan) |
| 14 | *Evodia glabrifolia* (Champ.) N. P. Balakr. | [Rutaceae](http://frps.eflora.cn/frps/Rutaceae) | Ledong (Hainan); Shanghang (Fujian) | Ledong (Hainan) | Ledong (Hainan) |
| 15 | *Fargesia fractiflexa*T.P.Yi | Gramineae | Mengla (Yunnan) | Jinping, Mengla (Yunnan) | —— |
| 16 | [*Firmiana platanifolia*(L.f.) Marsili](http://www.theplantlist.org/tpl1.1/record/kew-2813001) | [Labiatae](http://frps.iplant.cn/frps/Labiatae) | Xinshao (Hunan) | —— | Xinshao (Hunan) |
| 17 | [*Hedychium coronarium*J. Koenig](http://www.theplantlist.org/tpl1.1/record/kew-248115) | Zingiberaceae | Xinzhu (Taiwan) | —— | Xinzhu (Taiwan) |
| 18 | [*Indocalamus guangdongensis* H. R. Zhao & Y. L.Yang](http://www.theplantlist.org/tpl1.1/record/kew-420194) | Gramineae | Qingcheng, Haifeng (Guangdong); Tongzi (Guizhou) | Qingcheng, Haifeng (Guangdong) | Qingcheng, Haifeng (Guangdong) |
| 19 | *Indocalamus herklotsii* McClure | Gramineae | Pingle (Guangxi); Peixian (Hunan) | Pingle (Guangxi); Peixian (Hunan) | Pingle (Guangxi) |
| 20 | *Indocalamus latifolius* (Keng) McClure | Gramineae | Cangshan (Fujian); Haifeng, Huidong (Guangdong); Shangli, Dean (Jiangxi); Quanwan (Xianggang); Meilan (Hainan); Jiulongpo (Chongqing) | Haifeng, Huidong (Guangdong); Shangli (Jiangxi); Meilan (Hainan) | Haifeng, Huidong (Guangdong); Shangli (Jiangxi); Jiulongpo (Chongqing) |
| 21 | [*Indocalamus tessellatus*(Munro) Keng f.](http://www.theplantlist.org/tpl1.1/record/kew-420239) | Gramineae | Haifeng, Huidong (Guangdong); Dangzai, Datang (Macao); Lechang, Meilan (Hainan); Ningdu, Shangli (Jiangxi); Shanghang, Cangshan (Fujian); Pudong, Fengxian (Shanghai); Jiulongpo (Chongqing); Chongzhou (Sichuan) | Haifeng, Huidong (Guangdong); Ningdu (Jiangxi); Jiulongpo (Chongqing); Cangshan (Fujian); Lechang, Meilan (Hainan) | Haifeng, Huidong (Guangdong); Ningdu, Shangli (Jiangxi); Meilan (Hainan); Fengxian (Shanghai) |
| 22 | [*Livistona chinensis*(Jacq.) R.Br. ex Mart.](http://www.theplantlist.org/tpl1.1/record/kew-114913) | Palmae | Wenchang (Hainan); Jinping (Yunnan) | —— | Wenchang (Hainan); Jinping (Yunnan) |
| 23 | [*Magnolia officinalis*Rehder & E.H.Wilson](http://www.theplantlist.org/tpl1.1/record/kew-117741) | [Magnoliaceae](http://frps.iplant.cn/frps/Magnoliaceae) | Pingle, Yongfu (Guangxi) | Yongfu (Guangxi) | Yongfu (Guangxi) |
| 24 | *Miscanthus floridulus*(Labill.) Warb. ex K. Schum. & Lauterb. | Gramineae | Jingning (Zhejiang); Xiapu (Fujian) | Jingning (Zhejiang); Xiapu (Fujian) | Jingning (Zhejiang); Xiapu (Fujian) |
| 25 | *Monocladus amplexicaulis*Chia et al. | Gramineae | Pingxiang (Guangxi) | Pingxiang (Guangxi) | Pingxiang (Guangxi) |
| 26 | *Musa acuminata* Colla | Musaceae | Jingxi, Leye (Guangxi); Yongsheng (Yunnan) | Jingxi (Guangxi); Yongsheng (Yunnan) | Jingxi (Guangxi); Yongsheng (Yunnan) |
| 27 | [*Musa balbisiana*Colla](http://www.theplantlist.org/tpl1.1/record/kew-254762) | Musaceae | Lingshan, Jingxi (Guangxi); Shuangjiang (Yunnan); Wanning (Hainan) | Lingshan (Guangxi); Shuangjiang (Yunnan) | —— |
| 28 | [*Musa basjoo* Siebold & Zucc. ex Iinuma](http://www.theplantlist.org/tpl1.1/record/kew-254769) | Musaceae | Huidong, Haifeng (Guangdong); Leye, Jingxi (Guangxi); Hanjiang, Shanghang (Fujian); Taijiang, Xingren (Guizhou); Meilan (Hainan); Ledong, Meilan (Hainan); Guiyang (Hunan) | Huidong, Haifeng (Guangdong); Jingxi (Guangxi); Meilan (Hainan); Ledong, Meilan (Hainan); Guiyang (Hunan) | Huidong, Haifeng (Guangdong); Jingxi (Guangxi); Hanjiang, Shanghang (Fujian); Meilan (Hainan) |
| 29 | *Musa nana*Lour. | Musaceae | Huidong, Qingcheng (Guangdong); Ledong, Meilan (Hainan); Jingxi (Guangxi); Mengla (Yunnan) | Huidong, Qingcheng (Guangdong); Ledong (Hainan); Mengla (Yunnan) | Huidong, Qingcheng (Guangdong); Ledong (Hainan) |
| 30 | [*Musa sapientum* L.](http://www.theplantlist.org/tpl1.1/record/kew-254945) | Musaceae | Haifeng, Huidong (Guangdong); Jingxi (Guangxi) | Haifeng, Huidong (Guangdong) | Haifeng, Huidong (Guangdong); Jingxi (Guangxi) |
| 31 | *Musa itineras* Tutcher | Musaceae | Taijiang (Guizhou); Huanjiang (Guangxi) | Huanjiang (Guangxi) | Huanjiang (Guangxi) |
| 32 | [*Nelumbo nucifera* Gaertn.](http://www.theplantlist.org/tpl1.1/record/kew-2384945) | Nymphaeaceae | Huidong, Haifeng (Guangdong); Lingshui, Meilan (Hainan); Peixian (Jiangsu); Zhenyuan (Gansu) | —— | Huidong, Haifeng (Guangdong); Peixian (Jiangsu) |
| 33 | [*Pandanus* *austrosinensis* T. L.Wu](http://www.theplantlist.org/tpl1.1/record/kew-285867) | Pandanaceae | Ledong, Lingshui (Hainan) | —— | Lingshui (Hainan) |
| 34 | *Pandanus tectorius* Parkinson ex Du Roi | Pandanaceae | Ledong, Lingshui (Hainan); Leizhou, Sanxiang (Guangdong) | Ledong, Lingshui (Hainan); Leizhou (Guangdong) | Ledong, Lingshui (Hainan); Leizhou (Guangdong) |
| 35 | [*Perilla frutescens*(L.) Britton](http://www.theplantlist.org/tpl1.1/record/kew-150299) | [Labiatae](http://frps.iplant.cn/frps/Labiatae) | Fuxin, Yinzhou (Liaoning) | —— |  |
| 36 | [*Phragmites* *australis* (Cav.) Trin. ex Steud.](http://www.theplantlist.org/tpl1.1/record/kew-433921) | Gramineae | Decheng, Dongchangfu (Shandong); Yongqiao, Huaining (Anhui); Haidian, Shunyi (Beijing); Hexi (Tianjin); Cixian, Xianghe, Suning (Hebei); Xiaoyi, Ruicheng (Shanxi); Chengxi (Qinhai); Shuanta, Yinzhou (Liaoning) | Haidian (Beijing); Dongchangfu (Shandong); Ruicheng (Shanxi) | Haidian (Beijing); Huaining (Anhui); Dongchangfu (Shandong); Ruicheng (Shanxi); Yinzhou (Liaoning) |
| 37 | *Phrynium* *capitatum* Willd. | Marantaceae | Deqing (Guangdong); Meilan (Hainan); Leye, Jingxi (Guangxi) | Deqing (Guangdong); Jingxi (Guangxi) | Deqing (Guangdong); Meilan (Hainan); Jingxi (Guangxi) |
| 38 | *Phrynium hainanense* T. L. Wu & S. J. Chen | Marantaceae | Wanning (Hainan) | —— | Wanning (Hainan) |
| 39 | *Phrynium placentarium* (Lour.) Merr. | Marantaceae | Deqing (Guangdong); Jingxi (Guangxi) | —— | Deqing (Guangdong); Jingxi (Guangxi) |
| 40 | *[Phyllostachys](http://www.theplantlist.org/tpl1.1/record/kew-434065)**[bambusoides](http://www.theplantlist.org/tpl1.1/record/kew-434065)*[Siebold & Zucc.](http://www.theplantlist.org/tpl1.1/record/kew-434065) | Gramineae | Pingle, Huanjiang (Guangxi); Taijiang (Guizhou)；Shanghang (Fujian) | —— | Pingle, Huanjiang (Guangxi); Mengla (Yunnan) |
| 41 | [*Phyllostachys heteroclada*Oliv.](http://www.theplantlist.org/tpl1.1/record/kew-434162) | Gramineae | Maguan (Yunnan); Pingle (Guangxi) | —— | Maguan (Yunnan) |
| 42 | [*Phyllostachys heterocycla*(Carrière) Matsum.](http://www.theplantlist.org/tpl1.1/record/kew-434168) | Gramineae | Yuqing (Guizhou); Lushi (Henan) | —— | Lushi (Henan) |
| 43 | *Piper sarmentosum*Roxb. | [Piperaceae](http://frps.iplant.cn/frps/Piperaceae) | Leizhou, Deqing (Guangdong), Pingle (Guangxi) | Leizhou, Deqing (Guangdong) | Leizhou, Deqing (Guangdong), Pingle (Guangxi) |
| 44 | [*Pleioblastus amarus*(Keng) Keng f.](http://www.theplantlist.org/tpl1.1/record/kew-434682) | Gramineae | Eshan (Yunnan) | —— | Eshan (Yunnan) |
| 45 | *Podocarpus nagi*(Thunb.) Pilg. | [Podocarpaceae](http://frps.eflora.cn/frps/Podocarpaceae) | Lingshan (Guangxi), Leizhou (Guangdong) | —— | Lingshan (Guangxi) |
| 46 | [*Quercus dentata* Thunb.](http://www.theplantlist.org/tpl1.1/record/kew-172460) | Fagaceae | Lushi, Xiping (Henan); Baishui (Shanxi); Pingyi (Shandong); Yanggao (Shanxi) | Lushi, Xiping (Henan); Pingyi (Shandong) | Lushi, Xiping (Henan); Baishui (Shanxi); Pingyi (Shandong) |
| 47 | *Rhapis excelsa*(Thunb.) Henry | Palmae | Jinping, Mengla (Yunnan) | —— | Jinping (Yunnan) |
| 48 | [*Saccharum* *officinarum* L.](http://www.theplantlist.org/tpl1.1/record/kew-439977) | Gramineae | Sanxiang, Deqing (Guangdong) | —— | Deqing (Guangdong) |
| 49 | [*Sorghum bicolor*(L.) Moench](http://www.theplantlist.org/tpl1.1/record/kew-443283) | Gramineae | Xiangyang (Hubei); Dongchangfu (Shangdong) | —— | Xiangyang (Hubei) |
| 50 | [*Sterculia* *nobilis* Sm.](http://www.theplantlist.org/tpl1.1/record/kew-2580138) | [Sterculiaceae](http://frps.eflora.cn/frps/Sterculiaceae) | Haizhu, Sanxiang (Guangdong), Jingxi (Guangxi) | —— | Haizhu, Sanxiang (Guangdong) |
| 51 | [*Terminalia catappa* L.](http://www.theplantlist.org/tpl1.1/record/kew-2431102) | [Myrtiflorae](http://frps.iplant.cn/frps/Myrtiflorae) | Xinzhu (Taiwan) | —— | Xinzhu (Taiwan) |
| 52 | *Thysanolaena maxima* (Roxb.) Kuntze | Gramineae | Jingxi (Guangxi); Qingcheng (Guangdong); Jinping, Mengla (Yunnan) | Jingxi (Guangxi); Qingcheng (Guangdong); Jinping (Yunnan) | Jingxi (Guangxi); Qingcheng (Guangdong) |
| 53 | [*Tilia* *tuan* Szyszył.](http://www.theplantlist.org/tpl1.1/record/tro-32200864) | Tiliaceae | Yanqing (Beijing) | —— | Yanqing (Beijing) |
| 54 | *Trachycarpus fortunei* (Hook.) H. Wendl. | Palmae | Qidong (Hunan); Xiaoting (Hubei) | —— | Qidong (Hunan); Xiaoting (Hubei) |
| 55 | *Vernicia* *fordii* (Hemsl.) Airy Shaw | Euphorbiaceae | Guiyang (Hunan); Nanxi (Sichuan) | —— | Guiyang (Hunan); Nanxi (Sichuan) |
| 56 | [*Zea* *mays* L.](http://www.theplantlist.org/tpl1.1/record/kew-450362) | Gramineae | Xiaoting (Hubei); Zhuchengshi, Decheng (Shangdong); Huangzhong, Chengxi (Qinhai); Huining (Gansu); Keerqin (Inner Mongolia); Yian (Heilongjiang); Tianshan (Xinjiang) | —— | Hongshan (Hubei); Huining (Gansu); Kuche (Xinjiang); Nanchuang (Chongqing); Yian (Heilongjiang) |
| 57 | *Zizania* *latifolia* (Griseb.) Turcz. ex Stapf | Gramineae | Wuzhong (Jiangsu) | —— | —— |

Area 1, 2, and 3 mean the areas where people characterize the flavor contributions, antiseptic functions, and the medicinal values of ZLs, respectively.
